# Exploring the Link between the Components of Metabolic Syndrome and the Risk of Depression

**DOI:** 10.1155/2015/586251

**Published:** 2015-12-07

**Authors:** Fang-Yih Liaw, Tung-Wei Kao, Ju-Ting Hsueh, Yi-Hsin Chan, Yaw-Wen Chang, Wei-Liang Chen

**Affiliations:** ^1^Division of Family Medicine, Department of Family and Community Medicine, Tri-Service General Hospital, National Defense Medical Center, Taipei, Taiwan; ^2^Division of Geriatric Medicine, Department of Family and Community Medicine, Tri-Service General Hospital, National Defense Medical Center, Taipei, Taiwan; ^3^Graduate Institute of Medical Sciences, National Defense Medical Center, Taipei, Taiwan

## Abstract

*Background.* Metabolic syndrome (MetS) has been reported with an increased risk of depression. MetS was also associated with insulin resistance. This study aimed to evaluate whether MetS components might contribute to depression in participants with insulin resistance (IR) or not.* Methods.* This study included 3,331 participants ≥18 years in the NHANES 2009-2010. Depressive symptoms were assessed using the Patient Health Questionnaire-9 (PHQ-9). MetS components were measured using blood chemistry and body measurements. IR was identified using the homeostasis model assessment method.* Results.* Predicted PHQ-9 scores significantly increased as the number of MetS components increased in patients with IR. The adjusted *β* coefficients of the predicted PHQ-9 score with 2, 4, and 5 MetS components were 1.803, 2.081, and 3.048, respectively (*P* for trend < 0.05). Low HDL-C levels were significantly associated with higher predicted total PHQ-9 scores in fully adjusted models in the IR group (*P* < 0.05).* Conclusion.* The results indicated that the presence of a greater number of components of MetS was significantly associated with higher predicted total PHQ-9 scores in participants with IR. Among the components of MetS, the most apparent association was observed between low HDL and higher predicted total PHQ-9 scores.

## 1. Introduction

Depression, which is associated with excessive mortality and disability, is expected to become the second highest cause of disability by 2020 [1]. In addition, metabolic syndrome (MetS) is prevalent in the general population and is an important factor in all-cause mortality and cardiovascular disease. Depending on the different definition of the syndrome used, composition (age, sex, ethnicity, and race) of the population studied, the urban or rural environment, and the region, worldwide prevalence of MetS ranges from <10% to 67% [2]. MetS is a cluster of cardiovascular disease risk factors, including central obesity, elevated blood pressure, hypertriglyceridemia, hyperglycemia, and decreased high density lipoprotein cholesterol (HDL-C).

Insulin resistance (IR) is a stage of prediabetes that has been implicated in the cause of MetS and is defined as declined sensitivity of the peripheral insulin receptors to the action of insulin. People with depression reportedly exhibit glucose intolerance and IR, and both IR [3] and MetS [4] have a bidirectional association with depression. Participants with MetS have higher prevalence of depression in comparison with those without MetS [4, 5]. 40%–60% patients with depression present hyperactive hypothalamic-pituitary-adrenal axis. Disruption of glucoregulatory mechanisms may lead to insulin resistance [6]. Therefore, IR could be a possible mechanism for the association between depression and MetS [7, 8]. Few attempts have been made to establish a direct relationship between depression and metabolic components in IR group. Therefore, we hypothesize that the presence of a greater number of features of metabolic syndrome would be associated with depressive score in participants with IR. To determine this, we investigate the relationship between MetS components and depression in IR and non-IR group by analyzing the data in the reports of the National Health and Nutrition Examination Survey 2009-2010.

## 2. Materials and Methods

### 2.1. Study Population

The data for this study is derived from the National Health and Nutrition Examination Survey (NHANES) 2009-2010. The NHANES is a nationally representative health survey of noninstitutionalized US citizens conducted by the National Center for Health Statistics (NCHS) of the Centers for Disease Control and Prevention (CDC). The NHANES included a stratified multistage probability design with planned oversampling of certain age and minority groups. Trained study staff conducted surveys with participants, and standard medical examinations were completed at mobile centers for the following information: height, weight, blood pressure, waist circumference, plasma lipid levels, and plasma glucose levels.

The overall response numbers for the 2009-2010 survey was *N* = 10,537. We excluded participants < 18 years (*N* = 4,010). Then, we excluded participants for whom data were lacking regarding the components of metabolic syndrome (*N* = 380), the household interview (*N* = 815), the results of laboratory and clinical examinations (*N* = 1,665), and the depressive score (*N* = 336). The result is in 3,331 eligible subjects (1607 men, 1724 women) with complete information. The NCHS Institutional Review Board approved the NHANES 2009-2010 study, and informed consent was acquired from participants prior to the study.

### 2.2. Measures

Depressive symptoms were assessed using the Patient Health Questionnaire-9 (PHQ-9), which is one of the most commonly used instruments for screening depression [9] and records the frequency of depressive symptoms over the previous 2 weeks using 9 items. The final question assesses the overall impairment from symptoms of depression. Responses are scored from 0 to 3, representing “not at all,” “several days,” “more than half the days,” and “nearly every day,” respectively, with total scores ranging from 0 to 27. Scores ≥ 10 are commonly used to define depression in clinical studies. The PHQ-9 is valid and easy to complete and can be completed in 2–5 minutes. The PHQ-9 provides a simple way to evaluate both diagnostic criteria and severity with a single, well-validated instrument [10].

Consistent with the revised National Cholesterol Education Program's Adult Treatment Panel III (NCEP ATP III), MetS was defined as the presence of ≥3 of the following characteristics: (1) abdominal obesity: waist circumference ≥ 102 cm in men and ≥88 cm in women; (2) high triglycerides: ≥150 mg/dL or patients who currently use lipid-lowering medications; (3) low HDL-C: <40 mg/dL in men and <50 mg/dL in women or patient on specific drug treatment; (4) high blood pressure: systolic blood pressure ≥130 mmHg or diastolic blood pressure ≥85 mmHg or current use of antihypertensive drugs; and (5) high fasting glucose: ≥110 mg/dL or current use of insulin or oral diabetic medications [11].

Chemical analysis of triglycerides and HDL-C (Roche Modular P Chemistry Analyzer, Indianapolis, IN, USA) was conducted by the Lipoprotein Analytical Laboratory at the University of Minnesota. Low density lipoprotein cholesterol levels were calculated using the Friedewald formula [12]. Serum C-reactive protein (CRP) levels were determined using latex-enhanced nephelometry. The other biochemistry profiles were analyzed using Beckman Synchron LX20 and Beckman UniCel DxC800 Synchron, Beckman Coulter Inc., Fullerton, CA.

All of the protocols used standardized methods with documented accuracy with respect to CDC reference methods [13]. The homeostasis model assessment of insulin resistance (HOMA-IR) was calculated using the following formula: HOMA-IR = fasting plasma glucose (mg/dL) *∗* fasting insulin (uIU/mL)/405 [14]. Based on previous studies [15, 16], a cutoff of 2.5 was chosen for HOMA-IR to identify IR.

The participants collected information on age, sex, race/ethnicity, body measurements (including height, weight, and waist circumference), blood pressure, and medical conditions in Mobile Examination Centers. Body mass index (BMI) was calculated by dividing the participant's weight in kilograms by the square of their height in meters (kg/m^2^). Trained NHANES staff measured waist circumference using standard protocols. Smoking status was based on lifetime use of ≥100 cigarettes. Alcohol use was defined as having at least 12 drinks in the past year. Coronary heart disease and stroke were based on self-report of physician diagnosis. Vigorous work activity was defined based on a response of participation in vigorous-intensity activity. Detailed specimen collections and processing instructions are provided in the NHANES Laboratory Procedures Manual, which is available on the NHANES website [13].

### 2.3. Statistical Analysis

SPSS (v18.0 for Windows; SPSS Inc., Chicago, IL, USA) was used to perform statistical analyses. Predicted values of total PHQ-9 scores were divided into non-IR and IR groups. The Chi-square test was used with categorical data and Mann-Whitney *U* test was used with continuous data. Then, the associations between PHQ-9 scores and the number of MetS components or each individual MetS component were determined. Based on previous studies, influential demographic factors and clinical standpoints were used as covariate adjustments. Multiple linear regression models were constructed for evaluation. We used 3 models with progressive degrees of adjustment. Model 1 was adjusted for age, sex, and race/ethnicity. Model 2 was further adjusted for BMI, alcohol use, smoking status, coronary heart disease, stroke, and vigorous working activity. Model 3 was further adjusted for serum CRP, serum total bilirubin, serum uric acid, and HOMA-IR. The *P* values for trend tests were determined by treating the components of MetS as continuous variables (1–5) to observe the associations with increasing numbers of MetS components and total PHQ-9 score.

## 3. Results

The characteristics of the 3,331 participants (1607 men, 1724 women) stratified by presence of IR are summarized in Table 1. The prevalence of IR was 57.3%. Participants with IR had borderline significant higher rate of depression (8.4% versus 6.8%, *P* = 0.057). Participants with IR had significantly higher PHQ-9 scores (*P* = 0.003).

In the participants with IR, body matrix index (BMI), serum glucose levels, blood pressure, waist circumference, serum triglycerides, serum uric acid levels, CRP levels, and serum glucose levels were significantly higher, and HDL-C was significantly lower compared with the non-IR group (all, *P* < 0.05).

**Figure 1:**
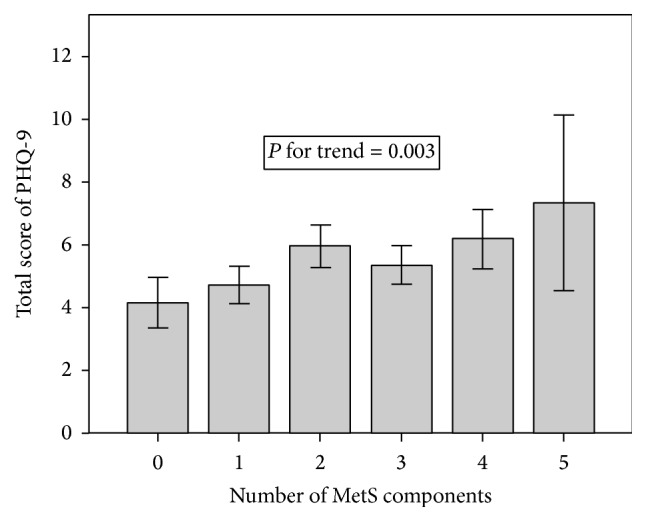
Mean values of total score of PHQ-9 across the number of MetS components in IR group.

Results from the models examining the association between the number of metabolic components and the predicted PHQ-9 values stratified by IR are presented in Table 2. In the IR group, there was a strong linear increase in the predicted PHQ-9 score with an increase in the number of MetS components (Figure 1, *P* for trend = 0.003). After additional adjustment, the *β* coefficients of the predicted PHQ-9 score with 2, 4, and 5 MetS components were 1.803, 2.081, and 3.048, respectively (*P* for trend <0.05). In the IR group, low HDL-C was significantly associated with higher predicted total PHQ-9 scores in fully adjusted models (*P* < 0.05) (Table 3). Other MetS components were not significantly associated with PHQ-9 scores.

## 4. Discussion

By using the MetS definition from the NCEP:ATP III criteria, we examined the association between MetS components and depressive symptoms among US adults. Insulin resistance was identified as a mediator in the relationship between depressive symptoms and MetS. To the best of our knowledge, this is the first large population-based study including community-dwelling adults to examine this association using PHQ-9, HOMA-IR, and numbers of MetS components. The findings indicate that the severity of depressive symptoms, as assessed by the PHQ-9, was higher with a greater number of MetS components in subjects with IR compared to those without it, independent of age, sex, race, BMI, smoking status, alcohol use, presence of comorbidities, serum CRP, total bilirubin, serum uric acid, and HOMA-IR levels. Furthermore, low HDL-C, as a single MetS component, was significantly associated with increasing PHQ-9 scores.

Depression is comorbid in a variety of disorders, including chronic obstructive pulmonary disease, cardiovascular disease, and diabetes, and depressive disorders have been associated with all-cause mortality [17]. Previous studies have also indicated that depression may be related to MetS and adverse health outcomes [18]. A systematic review of observational studies showed a link between depression and MetS [4]. Most studies only provided data on the prevalence of depression in participants with MetS compared with those without. Another systemic review represents small but significant association between depression and IR [19]. In the present study, the PHQ-9 score was significantly associated with MetS components in the IR group, but not in the non-IR group. Pearson et al. found that the depressive disorder, assessed by DSM-IV criteria, was significantly related to IR as indexed by HOMA in young adults [6]. Okamura et al. speculated that patients with depression also had higher IR and that IR-associated abnormalities could be resolved after resolution of depression [20]. However, other studies have resulted in conflicting results, including a study in Swedish women with a risk of diabetes mellitus, in which self-rated symptoms of depression were not related to IR [21]. In a cross-sectional analysis of 4286 British women aged 60–79 years, IR was inversely associated with depression [22]. The patients with higher serotonin concentrations are less likely to have depressive moods, indicating that insulin sensitivity may be related to tryptophan metabolism [23].

Although the exact definition differs by expert group and organization, one of the most commonly used definitions of MetS is based on the revised NCEP:ATP III. However, It is recognized that MetS features chronic inflammation characterized by abdominal obesity, hypertension, hyperglycemia, and dyslipidemia. Inflammation is associated with markers of IR, obesity, and MetS [24], and inflammation has also been implicated in the etiology of depressive disorders in the form of immune dysregulation [25] and activation of the inflammatory response system. Patients with major depressive disorders have higher levels of proinflammatory cytokines, such as interleukin-6, interleukin-1, and tumor necrosis factor-alpha, than the general population [26, 27]. In an analysis of the 2009-2010 NHANES dataset, patients with depression had higher levels of CRP [28]. Therefore, inflammation might be an underlying link between MetS and depression. In a population-based health survey of noninstitutionalized US citizens conducted between 1988 and 1994 [29], the prevalence of MetS was higher in young women with depression. In the present study, patients in the IR group with a greater number of MetS components had higher predicted PHQ-9, even after adjusting for confounding factors, such as CRP. Therefore, it is possible that IR has a more important role in the symptoms of depression in subjects with greater number of MetS components. Otherwise, MetS might contribute to more depressive symptoms in patients with IR.

Mood disorders are associated with abnormalities of the hypothalamic-pituitary-end organ axes [30]; a hyperactive hypothalamic-pituitary-adrenal axis occurs in 40–60% of patient with major depressive disorders [31]. Patients with cortisol hypersecretion have high rates of depression [32] and IR [33]. Excess circulating cortisol disrupts glucoregulatory mechanisms and induces hyperinsulinemia and IR. Furthermore, abnormal serotonergic function in the central nervous system influences the pathogenesis of depression [34]. Selective serotonin reuptake inhibitors, which are antidepressant drugs, can improve IR in patients with obesity and type 2 diabetes mellitus [35]. Alternatively, people with depression are typically physically inactive, have poor dietary habits, smoke, lead a sedentary lifestyle, and have poor compliance with medical treatment, which can increase the risk of IR and MetS and ultimately lead to cardiovascular disease and type 2 diabetes mellitus [36, 37].

In the present study, low HDL-C was corrected with higher depression scores. A previous study reported that low HDL-C is consistently linked to depression and suicidal behavior [38]. A prospective study regarding the 5-year probability of depression in older men showed that the risk of depression increases as the plasma HDL-C concentration decreases [39]. The pathophysiology underlying this association remains unclear. Low HDL-C levels are associated with coronary heart disease; HDL-C has a role in reversing cholesterol transport and anti-inflammation. Because of the pleiotropic properties, including antioxidative function and suppression of monocyte and lymphocyte activity, serum HDL-C levels might play a role in depression [40]. Therefore, inflammation might be an early event in high depression scores in individuals with low HDL-C levels.

A limitation of the present study is that the self-reported PHQ-9 was used instead of structured diagnostic scales such as the DSM-IV to identify depression, which might affect the validity of the findings. Second, the cross-sectional nature of the survey resulted in measurement of HOMA-IR, PHQ-9, and MetS components at a single time rather than with repeated long-term observations. Thus, further prospective longitudinal studies should be conducted.

The presence of a greater number of features of MetS is associated with higher risk of depressive symptoms only in participants with IR. The association between depressive symptoms and MetS may possibly be mediated by IR. Improved IR and MetS components might ameliorate depressive symptoms and decrease the risk of cardiovascular disease [37] and type 2 diabetes mellitus [41]. Our findings highlight that depression scores are associated with more MetS components and low HDL-C levels in patients with IR in the United States. In patients with IR, MetS components might play a role in more depressive symptoms. Therefore, more attention should be given to MetS components and IR in the context of depression. Future studies should also investigate the relationships between IR, MetS, and depression, as well as the major biological mechanisms related to depression.

## Figures and Tables

**Table 1:** Characteristics of participants with or without metabolic syndrome.

Variables	Insulin resistance *N* = 1909	Noninsulin resistance *N* = 1422	*P* value
Continuous variables			
Age (years), mean (SD)	43.95 (20.93)	42.99 (20.46)	0.18
BMI (kg/m^2^), mean (SD)	30.82 (7.26)	24.83 (4.78)	<0.001
Systolic blood pressure, mean (SD)	121.87 (18.02)	118.37 (18.27)	<0.001
Diastolic blood pressure, mean (SD)	67.66 (13.94)	66.1 (12.63)	0.001
Waist circumference (cm), mean (SD)	102.55 (17.01)	87.59 (12.874)	<0.001
LDL (mg/dL), mean (SD)	111.59 (35.3)	109.91 (34.73)	0.174
Serum triglycerides (mg/dL), mean (SD)	142.31 (109.47)	97.94 (96.35)	<0.001
HDL (mg/dL), mean (SD)	49.04 (13.87)	59.83 (15.69)	<0.001
C-reactive protein, mean (SD)	0.45 (0.83)	0.29 (0.79)	<0.001
Serum glucose (mg/dL), mean (SD)	111.91 (34.83)	95.26 (15.93)	<0.001
Serum bilirubin (mg/dL), mean (SD)	0.76 (0.28)	0.84 (0.31)	<0.001
Serum uric acid (mg/dL), mean (SD)	5.68 (1.45)	5.05 (1.31)	<0.001
PHQ-9, mean (SD)	5.5 (5.05)	4.81 (4.28)	0.003
Categorical variables^c^			
Male, *n* (%)	982 (51.4)	625 (44)	<0.001
Non-Hispanic white, *n* (%)	774 (40.5)	716 (50.3)	<0.001
CHD, *n* (%)	73 (3.8)	39 (3.3)	0.195
Stroke, *n* (%)	68 (3.6)	26 (1.8)	0.009
Alcohol, *n* (%)	981 (51.3)	808 (56.8)	0.004
Smoke, *n* (%)	706 (36.9)	510 (35.8)	0.15
Vigorous work activity, *n* (%)	308 (16.1)	227 (15.9)	0.68
Score of PHQ-9 >10, *n* (%)	160 (8.4)	98 (6.8)	0.057

BMI: body mass index; LDL: low density lipoprotein; HDL: high density lipoprotein; CRP: C-reactive protein; CHD: coronary heart disease; SD: standard deviation.

**Table 2:** Regression coefficients of number of metabolic syndromes for PHQ-9 score predicted.

Variables	Insulin resistance (HOMA-IR ≥ 2.5)						Noninsulin resistance (HOMA-IR < 2.5)					
	Model 1		Model 2		Model 3		Model 1		Model 2		Model 3	
	*β* (95% CI)	*P* value	*β* (95% CI)	*P* value	*β* (95% CI)	*P* value	*β* (95% CI)	*P* value	*β* (95% CI)	*P* value	*β* (95% CI)	*P* value
Number of MetS components												
1	0.972 (**−**0.5891, 2.533)	0.222	0.788 (−0.799, 2.376)	0.33	0.773 (−0.822, 2.367)	0.342	0.628 (−0.179, 1.435)	0.127	0.622 (−0.216, 1.461)	0.146	0.578 (−0.266, 1.422)	0.179
2	2.102 (0.586, 3.618)	0.007	1.788 (0.204, 3.372)	0.027	1.803 (0.23, 3.394)	0.026	0.512 (−0.425, 1.448)	0.284	0.395 (−0.629, 1.420)	0.449	0.205 (−0.861, 1.272)	0.705
3	1.755 (0.206, 3.304)	0.026	1.405 (−0.227, 3.037)	0.092	1.396 (−0.247, 3.038)	0.096	0.581 (−0.926, 2.089)	0.449	0.312 (−1.32, 1.944)	0.708	0.051 (−1.623, 1.725)	0.952
4	2.477 (0.88, 4.153)	0.004	2.153 (0.392, 3.914)	0.017	2.081 (0.289, 3.873)	0.023	0.143 (−2.658, 2.944)	0.920	0.065 (−2.775, 2.905)	0.964	−0.109 (−2.962, 2.743)	0.940
5	3.444 (1.145, 5.744)	0.003	3.202 (0.816, 5.588)	0.009	3.048 (0.638, 5.459)	0.013	3.204 (−2.824, 9.231)	0.297	2.519 (−2.321, 2.19)	0.410	1.886 (−4.239, 8.010)	0.546
*P* for trend	0.001		0.004		0.008		0.236		0.495		0.784	

Model 1 = age, sex, race/ethnicity.

Model 2 = Model 1 + (BMI, alcohol drinking, smoking, coronary heart disease, stroke, and vigorous working activity).

Model 3 = Model 2 + (serum C-reactive protein, serum total bilirubin, serum uric acid, and HOMA-IR).

**Table 3:** Regression coefficients of components of metabolic syndrome for PHQ-9 score predicted.

Components of metabolic syndrome	Insulin resistance (HOMA-IR ≥ 2.5)						Noninsulin resistance (HOMA-IR < 2.5)					
	Model 1		Model 2		Model 3		Model 1		Model 2		Model 3	
	*β* (95% CI)	*P* value	*β* (95% CI)	*P* value	*β* (95% CI)	*P* value	*β* (95% CI)	*P* value	*β* (95% CI)	*P* value	*β* (95% CI)	*P* value
Abdominal obesity	0.056 (−0.157, 1.525)	0.111	0.039 (−0.51, 1.469)	0.342	0.04 (−0.508, 1.48)	0.337	0.004 (−0.692, 0.768)	0.919	−0.003 (−2.091, 2.278)	0.952	−0.009 (−1.064, 0.911)	0.879
High blood pressure	0.037 (−0.382, 1.239)	0.299	0.042 (−0.331, 1.31)	0.243	0.037 (−0.386, 1.26)	0.298	0.039 (−0.485, 1.322)	0.363	0.038 (−0.498, 1.314)	0.37	0.035 (−0.526, 1.292)	0.409
High triglycerides	0.07 (0.06, 1.421)	0.033	0.057 (−0.076, 1.28)	0.082	0.052 (−0.145, 1.235)	0.121	0.027 (−0.576, 1.224)	0.480	0.011 (−0.781, 1.032)	0.785	0.003 (−0.889, 0.954)	0.945
Low HDL cholesterol	0.089 (0.249, 1.591)	0.007	0.075 (0.097, 1.448)	0.025	0.075 (0.096, 1.46)	0.025	0.028 (−0.529, 1.176)	0.457	0.013 (−0.709, 1.012)	0.729	0.001 (−2.095, 2.145)	0.988
High glucose	0.074 (0.041, 1.466)	0.038	0.069 (−0.004, 1.427)	0.051	0.059 (−0.134, 1.341)	0.108	−0.1 (−1.17, 0.902)	0.8	−0.14 (−1.205, 0.846)	0.731	−0.17 (−1.258, 0.814)	0.674

Model 1 = age, sex, race/ethnicity.

Model 2 = Model 1 + (BMI, alcohol drinking, smoking, coronary heart disease, stroke, and vigorous working activity).

Model 3 = Model 2 + (serum C-reactive protein, serum total bilirubin, serum uric acid, and HOMA-IR).
